# (Dis)Incentivizing Patient Satisfaction Metrics: The Unintended Consequences of Institutional Bias

**DOI:** 10.1089/heq.2018.0065

**Published:** 2019-02-04

**Authors:** Sylk Sotto-Santiago, James E. Slaven, Theresa Rohr-Kirchgraber

**Affiliations:** ^1^ Department of Medicine, Indiana University School of Medicine, Indianapolis, Indiana.; ^2^ Department of Biostatistics, Indiana University School of Medicine, Indianapolis, Indiana.

**Keywords:** gender, institutional bias, patient satisfaction, physician faculty, race/ethnicity, underrepresented

## Abstract

**Background:** Patient satisfaction surveys as a metric for quality-based financial incentives carry a risk of bias toward women and underrepresented physicians. Previous assessments in our department of medicine found that most women faculty were rated in the bottom quartile of patient satisfaction scores, whereas analysis of scores for underrepresented physicians had not been performed. To investigate, we compared patient satisfaction scores and relevant demographics of faculty physicians during 1 year when quality-related financial incentives were offered based on this metric.

**Methods:** Patient satisfaction and communication scores collected during academic year 2015–2016 were obtained for 369 physicians (119 women and 250 men) at Indiana University Health system. Independent variables included physician gender, race, ethnicity, and subspecialty or division; 190 physicians constituted the study cohort for whom data were available for comparison. Statistical analyses were performed to determine if there were differences between gender and race in patient satisfaction scores (mean, median, *t*-tests, and Chi-square tests). A factorial analysis of variance model was performed to incorporate both main effects and to determine if there was a significant interaction between them.

**Results:** Median and mean of scores were lower for women physicians and underrepresented physicians. Analysis demonstrated nonsignificant effect between gender-segregated cohorts. Racially underrepresented physicians had significantly lower mean scores than their white colleagues [*F*(4, 185)=2.46, *p*=0.046].

**Conclusion and Relevance:** Our results indicate a significant difference in patient satisfaction scores between underrepresented and white physicians. These data may suggest a potential bias, among patients and institutional practices, ultimately leading to pay inequities through differences in financial incentives toward underrepresented physicians.

## Introduction

The Agency for Healthcare Research and Quality (AHRQ) first released the Consumer Assessment of Healthcare Physicians and Systems Clinician & Group Survey (CG-CAHPS) for adults and children in 2007.^1^ This survey was designed to assess patients' experiences with physicians and health care staff, as a tool for improving the care provided by individual physicians, sites of care, medical groups, or provider networks.^1^ Organizations use CG-CAHPS data for several purposes, and many organizations are including this patient satisfaction data as a factor in productivity payments to physicians. Although designed to measure inpatient hospital-level performance, some health systems disaggregate their CAHPS data to compare, assess, and incentivize improvements in patient satisfaction and quality measures for individual physicians, nurses, and other staff. Some reports confirm this presently happening. A study by Tefera in 2016 suggested that because health systems have identified patient experience as a potential source of competitive advantage, such actions can create “perverse and harmful incentives to elicit positive survey responses.”^2^ The authors felt doing so is contrary to the survey's design and policy aim.^2^ Hospital CAHPS (H-CAHPS) is not suitable for evaluating or incentivizing individuals or groups within a hospital, as it is meant to assess the entire hospital experience and culture of patient centeredness.

As part of our ongoing quality improvement efforts, the department of medicine examined overall patient satisfaction and communication scores during academic year 2015–2016 in which quality-related patient satisfaction financial incentives were offered to physician faculty members. The outpatient satisfaction component of the total quality incentive was weighted at ≥30% of the total performance-based bonus. Physicians with scores below the 30th percentile lost their entire quality incentive ($10,000 maximum). Anecdotally, women faculty in certain subspecialties were rated in the bottom quartile of patient satisfaction scores, resulting in remediation efforts, including encouraged (and sometimes mandatory) participation in a system-sponsored physician communication program, and limiting their participation in additional quality-based financial incentives during the same incentive period.

Few studies have looked at the specific relationship between race or ethnicity and physicians' patient satisfaction scores. However, the literature does provide mixed views on the relationship between patient satisfaction and physician gender. In one study, 509 new adult patients were randomized to see male or female primary care physicians at an academic medical center outpatient facility.^3^ The results showed that patients perceived that female physicians spent a significantly greater portion of the visit on preventative services and counseling, whereas male physicians devoted more time to technical practice behaviors and discussions of substance abuse. In multivariate analysis adjusting for patient characteristics, patients of female physicians were more (27%) satisfied with their experience than were patients of male physicians.^3^ Examining patient-provider dyads does not appear to add more clarity to this issue. Among patients who choose their physician, women choosing female doctors were the least satisfied in four measures of satisfaction (physician communication, physician technical skills, physician focus on prevention, and overall satisfaction), whereas male patients of female physicians were the most satisfied. This suggests that patients who choose their physician may have different expectations, and the difficulty of fulfilling these expectations may present particular challenges for female physicians.^4^

Even when female physicians are shown to be more patient centered than their male counterparts, this does not necessarily translate into greater patient satisfaction. Researchers videotaped encounters between physicians and patients in a hospital, measured patient satisfaction, and used trained coders to rate patient centeredness.^5^ In the inpatient/emergency department setting, female physicians were determined to be more patient centered than were male physicians; however, patient centeredness associated more strongly in satisfaction among male than female physicians, suggesting that male physicians were given more “credit” for being patient centered than did female physicians.^6^

Implicit or unconscious biases involve associations outside conscious awareness that lead to a negative evaluation of a person on the basis of characteristics such as race or gender.^7,8^ A number of studies have focused on implicit bias in health care, mostly from the direction of provider to patient encounters, often addressing evidence that health care professionals display implicit biases toward patients.^8^ Unfortunately, similar studies addressing patient bias toward physicians are limited, but in the current US sociopolitical environment more physicians are describing bias, racist, and discriminatory behaviors by their patients.^9,10^ In addition, in the context of organizational structures, institutional bias is defined as established customs and practices that systematically reflect and produce group-based inequities.^11^ Concern regarding potential patient and institutional biases prompted this study; intended to determine whether patient satisfaction scores demonstrate possible bias toward or against certain physician groups, subsequently affecting a group's financial incentive payments.

## Methods

Our departmental review included faculty physicians primarily working at both the Indiana University (IU) Hospital and IU Methodist Hospital. Provider patient satisfaction quality-related incentives were based on components of the CG-CAHPS that directly related to physician interaction with the patient. CG-CAHPS survey sample questions pertinent to physicians included the following:

- How often did this provider explain things in a way that was easy to understand?
- How often did this provider listen carefully to you?
- How often did this provider seem to know the important information about your medical history?
- How often did this provider show respect for what you had to say?
- How often did this provider spend enough time with you?
- What number would you use to rate this provider?
- Would you recommend this provider's office to your family and friends?
- How often did you have confidence and trust in this provider?
- How often did you get as much information about your condition and treatment as you wanted from this provider?

This retrospective study examined satisfaction scores during academic year 2015–2016 from patients of 369 faculty members. Faculty physicians represented 12 divisions (cardiology, clinical pharmacology, gastroenterology, pulmonary and critical care, nephrology, infectious diseases, hematology and oncology, endocrinology, rheumatology, palliative and geriatrics, and general internal medicine). Patient satisfaction scores ranged from 50% to 100% with 100% as the maximum score. Survey included qualifier descriptors (never, sometimes, usually, and always) and overall rate of provider from 0 to 10, with 10 describing the best provider possible. The project was a departmental review/evaluation, no identifiable information was collected, and not deemed research with human subjects, hence not requiring review by IU Institutional Review Board.

We included patient-satisfaction data only of physician faculty members who had self-reported gender and race/ethnicity in our personnel database. No other data source exists for this information; hence individuals for whom this information was missing were excluded. We also believe in a process in which individuals are able to declare their own racial, ethnic, gender identity, and gender expression. Once this information was obtained, the data were stripped of names and other possible identifiable information, such as faculty rank and faculty appointment type. For physicians to be included we required availability of additional data points, including the number of patient satisfaction surveys reviewed (minimum of 5, the financial incentive requirement) and the accompanying overall patient satisfaction score.

Indiana University Health Physicians (IUHP) is the organization's branch responsible for collection of patient satisfaction information and owner of this data. IUHP sends a paper copy of the validated patient satisfaction survey to patients who had visited their facilities and seen by providers. Patients return completed surveys to a third party for processing and analysis of responses ensuring objectivity and reliability. The response rate is estimated at 26%. Data were obtained as an electronic spreadsheet (Microsoft Excel) from IUHP. Microsoft Excel Data Analysis Pack was utilized based on the data received. In addition, analyses were performed to determine if there were differences between gender and race in patient satisfaction scores. A factorial analysis of variance (ANOVA) model was performed to incorporate both main effects and to determine if there was a significant interaction between them. All analytic assumptions were verified and analyses were performed using SAS v9.4 (SAS Institute, Cary, NC). Scores were treated as continuous data. We compared gender (dichotomous), race/ethnicity (white, African American or black, Latino/a, Asian or Asian American, and multiracial), and internal medicine subspecialty/division using sample means and medians through unpaired *t*-tests, and ANOVA for significant relationships between racial/ethnic groups. Differences were considered significant at the *p*<0.05 level. All analytic assumptions were verified, including the linearity of the outcome variable, which allowed for the use of Student's *t*-test and ANOVA parametric tests. Although cell counts were adequate, Chi-square tests were verified with Fisher's exact tests.

## Results

A total of 369 physician faculty scores were reviewed. Of these, 190 met the inclusion criteria. Cohort characteristics are given in Table 1. Overall patient satisfactions scores ranged from 50% to 100%. The overall sample mean (standard deviation) score was 85.1% (9.7).

**Table 1.** **Patient Satisfaction Scores by Gender and Race**

Characteristic	Frequency (percentage)	Patient satisfaction score: mean (standard deviation)	*p*
Total participants	369; 190 Met inclusion criteria		
Gender^a^			
Women	48 (25.3)	84.5 (9.8)	0.310
Men	142 (74.7)	85.3 (9.7)	
Race^b^			
White	139 (73.2)	86.0 (9.5)	Reference
African American/black^c^	5 (2.6)	83.2 (9.5)	0.028
Latino/Hispanic^c^	3 (1.6)	77.5 (13.1)	0.042
Asian/Asian American	40 (21.0)	76.3 (11.3)	0.046
Multiracial	3 (1.6)	91.5 (2.0)	0.171

^a^ As self-reported.

^b^ Per personnel system.

^c^ Ethnicity information was also self-reported; however, this information has been excluded to further protect the confidentiality of physicians.

Using unpaired *t*-tests, gender-defined physician cohorts showed no significant differences in satisfaction scores (*p*=0.310) (Table 1). The effect size (Cohen's D) for this comparison is 0.082. *p*-Value from Chi-square tests indicated homogeneity of gender across race (*p*=0.674).

We additionally stratified physicians by subspecialties/divisions and compared within-strata gender-specific patient satisfaction scores (male physician vs. female physicians within each of the 12 divisions). Bivariate gender analysis through *t*-test indicated no statistically significant differences between any of the 12 divisions.

We also compared patient satisfaction scores based on race/ethnic groups as defined earlier. Each nonwhite self-identified racial/ethnic group scores were compared with those of their white colleagues. Scores for African American or black, Latino/a, and Asian or Asian American groups were significantly lower than for the white physician cohort (*p*=0.028, *p*=0.041, *p*=0.046, respectively). There was no significant difference between the white group and the multiracial group (*p*=0.171), although all results became nonsignificant after using a Bonferroni adjustment to control for inflated type I error rates on multiple pairwise comparisons (Table 1). Because of the small sample size within each of these race/ethnic groups, we also evaluated them as an underrepresented cohort. *p*-Value from *t*-test indicates that there is a significant difference between races; white physicians have slightly higher satisfaction scores (*p*=0.037). The effect size (Cohen's D) for this comparison is 0.340.

We performed ANOVA to study the effect among the different racial/ethnic groups and white group. Analysis demonstrated a statistical significant relationship for patient satisfaction scores at the *p*<0.05 level [*F*(4, 185)=2.46, *p*=0.046]. We also used two-way ANOVA to understand the overall interaction between gender and race on patient satisfaction. The results indicate nonsignificant interaction with the results being similar in the model without the interaction term.

Finally, the difference between white female physicians (*N*=34) and nonwhite women physicians (*N*=14) revealed the latter cohort had significantly lower patient satisfaction scores (*p*=0.030). We continued to look for stratification of gender and race in the interest of *intersectionality*, in acknowledgment that identity markers, such as that of women of color, do not exist independently of each other and creating complex convergence of bias and discrimination.^12^

## Discussion

Initially, the goal of this departmental review was to investigate the concern that women physicians could potentially be disadvantaged by a financial incentive focused on patient satisfaction scores. Through the lens of implicit bias and institutional bias we additionally questioned the data for underrepresented physician groups, subsequently also considering their financial incentive implications.

We found that underrepresented faculty physicians score significantly lower in patient satisfaction and that women of color also score significantly lower than their white female colleagues. Although previous literature had looked at patient satisfaction, this is the first departmental study, to our knowledge, to highlight the disparity among underrepresented faculty patient satisfaction scores and the connection to a disadvantage in financial incentives.^2–6^ This also appears to be the first study to explore patient satisfaction scores of underrepresented physicians in internal medicine specialties.

We considered the growing body of literature focused not only on implicit and institutional bias but also on the experiences of underrepresented faculty in academic medicine.^13–18^ They describe a “win or lose” academic medicine culture and institutional climate plagued with challenges, including those of bias, racism, and discrimination among others.^13^ This study should also be considered a contribution to that literature.

From its beginning in implicit social cognition, scholars demonstrated that awareness of stereotypes can affect judgment and behavior in relative independence from how an individual explicitly responds. Although at most academic medical centers, cultural competency education is part of the curriculum to improve care and reduce health inequities, we exhibit minimum efforts in addressing patient biases or discriminatory behaviors toward physicians.^10^ Furthermore, in the context of institutional bias, well-intentioned initiatives can have unexpected results. Although such financial incentives are encouraged and not driven by intentional bias, organizational decisions can contribute and perpetuate institutionalized discriminatory behaviors and bias. There are two important features of institutional bias. First, it can lead into favoritism of an advantaged group. Second, it can produce outcomes that cumulatively continue to benefit this advantaged group, even in the absence of overt racism or discrimination.^11^ Hence, tying financial incentives to patient satisfaction scores for the sake of quality may only hurt women and underrepresented physician groups.

Patient satisfaction scores reflect aspects of the visit that are not always under the physicians' control. The potential for the physician to feel “under siege” when the results are consistently below their peers and affect their income can lead to burnout, emotional distress, or the other extreme of disregarding all satisfaction survey results, thereby inhibiting the proposed goal of patient satisfaction surveys to improve patient–physician communication and enhance the patient experience.

Opportunities for further investigation of the role of patient bias and provider satisfaction could be strengthened by characterizing the demographics of the patient population served. Although this additional information could help shape the patient experience, the greatest accomplishment would be in understanding the role of the provider physician race/ethnicity in the patient's assessment of satisfaction and quality care. This study introduces the impact of policies and practices that although aimed to incentivize physicians, they could adversely affect total salary compensation of women and underrepresented groups. The goal of this communication is to encourage academic medicine and health care systems to study the impact and downstream effects of even the most positive policies. Our commitment is to disrupt structural systems that perpetuate not only the physician gender gap but also hinder progress in equity and inclusion.

## Limitations

Our study had several limitations. First, because of the sample size of our physician groups, it would be difficult to make explicit statements that this is the norm nationwide or that that bias is solely related to low patient satisfaction scores. It is important to reiterate that the importance of this review should be positioned within a case study, representative of the composition of the state and within the physician composition of IU Health. A nationwide study would provide broader sense of geographic location and demographics contributing to more comprehensive statements about the impact of provider gender and race/ethnicity on patient satisfaction. Second, without patient demographic information we are unable to control for the gender or race/ethnicity of patients and bias awareness. However, it is important to note that the academic health center is located among a population that is 85.4% white, 9.7% black, 7.0% Latino/Hispanic, and 2.4% Asian/Asian American.^19^ Third, although no statistical significance was found between genders and within specialties, it is important to note that only 12 internal medicine specialties were included in this study and as such, there might be instances in which gender differences, as well as race/ethnicity, could be more prevalent on other specialties.

## Conclusion

Understanding race-/ethnicity- and gender-based pay disparity that exists in medicine is important toward establishing equity. Any item that is brought forward as a financial incentive should be evaluated for its potential impact in creating larger financial gaps and supporting structural barriers that perpetuate equity and inclusion issues. Patient satisfaction scores can be influenced by many aspects and through important information, we would recommend that physician–patient communication and quality measures are evaluated in ways that consider potential impact on financial reimbursement. When bias is present, the potential impact to worsen the already significant pay discrepancy, along with the unconscious bias about the perception of competence of a physician, can be devastating.

## Abbreviations Used

ANOVA: analysis of variance
CG-CAHPS: Consumer Assessment of Healthcare Physicians and Systems Clinician & Group Survey
IU: Indiana University
IUHP: Indiana University Health Physicians
